# Citrulline, Intestinal Fatty Acid-Binding Protein and the Acute Gastrointestinal Injury Score as Predictors of Gastrointestinal Failure in Patients with Sepsis and Septic Shock

**DOI:** 10.3390/nu15092100

**Published:** 2023-04-27

**Authors:** Maciej Tyszko, Anna Lemańska-Perek, Jakub Śmiechowicz, Paulina Tomaszewska, Przemyslaw Biecek, Waldemar Gozdzik, Barbara Adamik

**Affiliations:** 1 Clinical Department of Anesthesiology and Intensive Therapy, Wroclaw Medical University, Borowska 213, 50-556 Wroclaw, Poland; 2 Department of Chemistry and Immunochemistry, Wroclaw Medical University, M. Sklodowskiej-Curie 48/50, 50-369 Wroclaw, Poland; 3 Faculty of Mathematics and Information Science, Warsaw University of Technology, Koszykowa 75, 00-662 Warsaw, Poland; 4 Faculty of Mathematics, Informatics and Mechanics, University of Warsaw, Banacha 2, 02-097 Warsaw, Poland

**Keywords:** citrulline, I-FABP, biomarker, sepsis, septic shock, intestinal injury, intensive care

## Abstract

Gastrointestinal (GI) failure can be both a cause of sepsis and a consequence of the systemic pro-inflammatory response in sepsis. Changes in biomarkers of enterocyte damage, citrulline and I-FABP (intestinal fatty acid binding protein), may indicate altered intestinal permeability and damage. The study group consisted of patients with sepsis (*N* = 28) and septic shock (*N* = 30); the control group included patients without infection (*N* = 10). Blood samples were collected for citrulline and I-FABP and a 4-point AGI score (acute GI injury score) was calculated to monitor GI function on days 1, 3, 5, 7, and 10. Citrulline concentrations in the study group were lower than in the control. Lower values were also noted in septic patients with shock when compared to the non-shock group throughout the study period. I-FABP was higher in the septic shock group than in the sepsis group only on days 1 and 3. Citrulline was lower in patients with GI failure (AGI III) when compared to AGI I/II, reaching significance on days 7 (*p* = 0.034) and 10 (*p* = 0.015); moreover, a higher AGI score was associated with an increased 28 day mortality (*p* = 0.038). The results indicate that citrulline measurements, along with the AGI assessment, have clinical potential in monitoring GI function and integrity in sepsis.

## 1. Introduction

Sepsis is defined as life-threatening organ dysfunction caused by a “dysregulated host response to infection” [1]. Gastrointestinal (GI) disorders are common in patients with sepsis and are associated with higher mortality [2]. GI complications can be both the cause of sepsis, as in patients with peritonitis, where the primary source of infection is the abdominal cavity, and the consequence of the systemic pro-inflammatory response observed in sepsis and septic shock. Changes to the intestinal-blood barrier over the course of sepsis are complex and include impaired regulation of splanchnic blood flow and intestinal mucosal hypoperfusion, increased permeability of the intestinal wall, coagulation-related local tissue perfusion disorders, and changes in the microbiome [3,4,5,6,7,8,9,10,11]. Another explanation for GI disorders may be disturbed intestinal motility in sepsis/shock as a result of the intensive use of sedatives and prolonged mechanical ventilation [12]. GI disorders in sepsis patients can have a multifaceted clinical presentation, including malnutrition, feeding intolerance, ulceration and bleeding, and bacterial translocation, all of which may contribute to worsening organ failure in sepsis and poor treatment outcome [13,14].

Diagnostics and monitoring of GI function in the intensive care unit (ICU) is a challenge for clinicians. Several scoring systems are routinely used to monitor the clinical condition of patients with sepsis, the most common being the Acute Physiology and Chronic Health Evaluation II (APACHE II), Sequential Organ Failure Assessment (SOFA), or the Simplified Acute Physiology Score (SAPS) score; unfortunately, the function of the GI system is not part of any of these scoring systems. Both an acute gastrointestinal injury (AGI) grading scheme and the recently developed Gastrointestinal Dysfunction Score (GIDS) score have been proposed for assessing the severity of GI disorders in ICU patients [2,15,16].

Therefore, the question arises as to whether biomarkers can be useful in monitoring the function of the GI tract in patients with sepsis as a method to support clinical assessment and scoring systems. Citrulline, a potential biomarker for assessing GI function in ICU patients, is mainly produced in humans by enterocytes in the small intestine and then released into the bloodstream; therefore, blood levels of citrulline may reflect the function and mass of intestinal epithelial cells [17]. Previous studies have shown that in critically ill patients, citrulline levels were often below the normal range of 30 to 50 mmol/mL; furthermore, low citrulline levels in this patient population correlated with clinical signs of GI dysfunction and failure, and with higher mortality [18,19,20,21]. Most studies evaluating changes in citrulline levels have been conducted in a general population of critically ill ICU patients; studies in the sepsis/septic shock population are rare and more research is needed in this category [20,22].

The second protein we studied as a potential biomarker of intestinal damage was I-FABP (intestinal fatty acid binding protein). It is a cytosolic protein specifically localized in the enterocytes of the small and large intestine. It is released into the bloodstream upon enterocyte destruction; therefore, I-FABP can be considered to be a marker of enterocyte function and intestinal mucosal injury [23]. Several studies have examined the association of I-FABP and citrulline with poor prognosis in sepsis, but the results have been inconclusive [24,25,26]. The results from our previous study showed that plasma levels of I-FABP, but not citrulline, were associated with significantly higher mortality in a group of patients with viral sepsis (COVID-19) [27].

The main objective of this study was to evaluate changes in citrulline and I-FABP plasma concentrations in a group of patients with bacterial sepsis. We hypothesized that shock-associated intestinal ischemia may induce damage of the intestinal barrier and functional enterocyte mass reduction. The effect of septic shock on intestinal cell integrity was studied using biomarker measurements and the current AGI classification system. The AGI score is a measure of GI disorders that can range from transient, partial impairment of GI function to permanent, long-term, and life-threatening damage. The relationship between AGI score and the biomarkers’ levels was assessed. A secondary objective was to study the predictive ability of ICU baseline citrulline and I-FABP levels for 28 day mortality.

## 2. Materials and Methods

From January to December 2019, a single-centre, prospective observational study of patients with sepsis or septic shock admitted to the ICU of the Wrocław University Hospital was conducted. The study protocol was accepted by the local Bioethical Committee (No. KB—822/2018). Written informed consent was obtained from the patient or a legally authorized representative. The study protocol complies with the 1975 Declaration of Helsinki, as revised in 1983.

### 2.1. Patients and Data Collection

The inclusion criteria for patients in the study group were the diagnosis of sepsis or septic shock upon admission to the ICU in accordance with the SEPSIS-3 definition and suspected or confirmed bacterial infection [1]. The exclusion criteria were as follows: pregnancy, terminal illness with no chance for meaningful recovery, expected ICU length of stay of 24 h or less, age <18 years old, and a history of previous severe gGI disabilities (chronic inflammatory diseases, such as ulcerative colitis, Lesniowski–Crohn disease, and viral hepatitis), chronic kidney failure, or pre-existing RRT dependency.

The primary sepsis management was conducted In accordance with the Surviving Sepsis Campaign guidelines [28]. Continuous renal replacement therapy (CRRT) was used as needed. On days 1, 3, 5, 7, and 10, clinical and laboratory data were recorded, and blood samples were taken from each patient. The Acute Physiology and Chronic Health Evaluation (APACHE) II score was used to assess patients’ baseline status. The Sequential Organ Failure Assessment (SOFA) score was used to monitor organ function each day. In patients with sepsis, these scores are commonly used as predictive tools in the ICU. APACHE II is a scale that includes both laboratory and clinical parameters (oxygenation status, cardiovascular assessment, consciousness, fever, essential electrolytes, complete blood count, and history of organ failure). The SOFA score focuses on the function of the vital systems: cardiovascular, respiratory, blood coagulation, liver, kidney, and the level of consciousness.

The Acute Gastrointestinal Injury (AGI) score was used to monitor GI function. The AGI score is a measure of GI failure in severe diseases, primarily evaluated in an ICU setting. As described by the ESICM Working Group on Abdominal Problems, the AGI score consists of 4 points [15]. Briefly,

1. AGI score I: (the patient is at risk of developing GI dysfunction) the function of the digestive tract is partially impaired; GI symptoms are associated with a known cause and transient;
2. AGI score II: (the patient developed GI dysfunction) the GI tract is unable to function properly to meet patient’s needs for nutrients and fluids;
3. AGI score III: (the patient developed GI failure) severe GI damage which does not respond to normal treatment and the general condition of the patient is not improving;
4. AGI score IV: (the patient developed GI failure with severe impact on distant organ function) persistent, long-term damage, resulting in worsening of multi-organ dysfunction syndrome or shock; life-threatening and requiring surgical intervention.

In our ICU practice, the AGI score is assessed daily to monitor GI function in patients with sepsis. Feeding intolerance (FI), described as high gastric residual volumes (GRV >500 mL), diarrhea (3 or more bowel movements with stool ≥200–300 g per day or volume >250 mL), or constipation (absence of bowel movements for 3 or more days), was recorded. The route of feeding administration (enteral/parenteral), type of nutrition product, and volume of feeding were recorded. In addition, 28-day mortality and length of stay in ICU and hospital were recorded.

### 2.2. Control Group

In order to compare the level of citrulline and I-FABP in ICU patients with and without sepsis, a control group was included in the study. The inclusion criteria in the control group were as follows: admission for first-time planned coronary artery bypass grafting under cardiopulmonary bypass, age >18 years old. The exclusion criteria in the control group were as follows: infectious complications after surgery, pre-existing RRT dependency, the need for RRT after surgery, ejection fraction <40%, and other co-morbidities involving diabetes and renal or liver failure. After the surgery, patients were routinely transferred to the ICU. In the control group, citrulline and I-FABP plasma concentrations were measured in blood samples collected on the first day of the ICU stay.

### 2.3. Sample Collection and Measurement of the Biomarkers

For plasma concentration measurements of I-FABP and citrulline, blood samples were collected (2.7 mL, tubes containing 0.109 M sodium citrate as an anticoagulant, BD Vacutainer, BD, NJ) through an arterial cannula routinely inserted at ICU admission. Samples were collected on days 1, 3, 5, 7, and 10, always in the morning. All patients (both fed enterally or receiving total parenteral nutrition through a central venous catheter) were infused with nutrients at a constant rate and there was no fasting period; as such, samples were taken while the patients were receiving food. Plasma separation was performed immediately after centrifugation for 10 min at 2000× *g*. The plasma was then stored at −70 °C. Commercial ELISA kits were used to analyze both I-FABP and citrulline levels (Quantikine ELISA Human FABP2/I-FABP Immunoassay, R&D Systems, Minneapolis, MN, USA; Human citrulline ELISA Kit, Cusabio Biotech Co., Ltd., Houston, TX, USA).

### 2.4. Statistical Analysis

The data were analyzed with Statistica 13 software (StatSoft, Inc., Tulsa, OK, USA) and R 3.6.01: R Core Team (2013). Continuous variables were summarized with three statistics: the median and the interquartile range between the 25th and 75th percentiles, and categorical as frequencies with percentages. The distribution of data was not normal based on the Shapiro–Wilk test and the analysis was performed with nonparametric tests. The Mann–Whitney U test was used to compare continuous variables between study groups at each time point. Categorical variables were analyzed using the Chi-square test, and contingency tables were used to analyze the frequency distribution of categorical variables. The predictive accuracy of the biomarkers measurements on admission to the ICU was tested using receiver operating characteristic curve (ROC) analysis, by calculating the area under the curve (AUC). The Youden’s statistic was used to select the optimum cut-off point for prognosis of GI failure (AGI III or AGI IV). Survival analysis of time to death was performed using the Kaplan–Meier curve and a log-rank test. The Kruskal–Wallis ANOVA by ranks was used for comparison of biomarker levels between subgroups with different AGI scores. The Friedman ANOVA was used to analyze within-group changes in performance overtime. *p*-values less than 0.05 were regarded as significant. The multivariable logistic regression analysis was performed to evaluate the association between values of the studied biomarkers and covariates and for the development of GI failure during ICU stay. The statistical metrics of the fitted model, including accuracy, sensitivity, specificity, and ROC AUC, were reported. The significance of predictors was analyzed considering the 95% confidence intervals and the resulting *p*-values.

## 3. Results

In the 12 month period, 58 septic patients met the inclusion criteria and were included in the analysis. Of these, 52% of patients had septic shock diagnosed on admission to the ICU, and 48% had sepsis. The median APACHE II score for the entire group was 25 points, the median SOFA score was 10 points, 71% of patients required mechanical ventilation on ICU admission, and 31% required continuous renal-replacement therapy (CRRT). The 28 day mortality was 38%. In order to compare the level of biomarkers in ICU patients with and without sepsis, a control group was included in the study. The control group consisted of ten adult patients after coronary artery bypass grafting, treated in the ICU after the surgery. Blood samples in the control group were collected on the day of admission to the ICU. The median age in the control group was 67 years (IQR 61–70 years), and males accounted for 70%. The septic group and the control group did not differ in terms of age (*p* = 0.828) and gender (*p* = 0.377). Immediately after surgery, all patients in the control group were mechanically ventilated, extubated, and breathing normally within 24 h of admission to the ICU. After extubation, all control group patients received a standard postoperative diet; there was no enteral tube feeding or parenteral feeding in this group. None of the patients in the control group developed infectious complications or kidney failure, and all patients survived. Patient characteristics are summarized in Table 1.

### 3.1. Levels of Citrulline and I-FABP in Septic Patients with and without Shock

First, the level of biomarkers measured in patients with sepsis and in the control group, i.e., patients without infection treated in the ICU, was compared. Blood samples in the control group were collected only once (day 1) and the concentrations of citrulline and I-FABP were compared with the values measured in the study group collected on day 1. The median citrulline level was significantly lower in the septic group when compared to the control group: 26.98 nmol/mL (IQR 21.45–32.28) vs. 39.82 nmol/mL (IQR 36.58–42.80), respectively, *p* < 0.001. The median I-FABP level was higher in the septic group than in the control group (719.66 pg/mL, (IQR 238.00–1346.78) vs. 545.67 pg/mL, (IQR 477.33–645.66), respectively), but the observed difference was not significant (*p* = 0.549). None of the patients in the control group developed infectious complications or organ failure during ICU treatment and all were transferred from the ICU within an average of 2 days (IQR 2.0–3.0).

**Figure 1:**
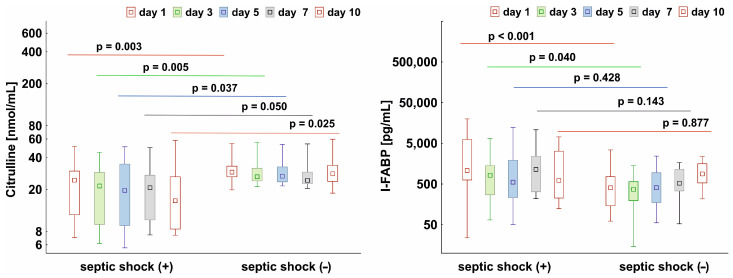
Graphs comparing the levels of Citrulline (**left**) and Intestinal Fatty Acid Binding Protein (I-FABP) (**right**) in the blood of patients with and without septic shock.

Next, to assess the effect of septic shock on intestinal cell damage, we compared the levels of biomarkers between septic patients with and without septic shock. Citrulline was significantly lower in patients with septic shock than in patients without shock on days 1, 3, 5, 7, and 10 (Figure 1, left panel). The median I-FABP concentrations were higher in the septic shock group than in the non-shock group, with statistically significant differences between the groups on day 1 and day 3 of the study (Figure 1, right panel).

As with other amino acids, some citrulline can be removed during renal replacement therapy; therefore, in patients with sepsis/septic shock, we compared citrulline levels between subgroups of patients who required/did not require continuous renal-replacement therapy (CRRT(+)/CRRT(−)). CRRT was used in 31% of patients to support renal function on admission to the ICU. Median citrulline levels were similar in the CRRT(+) and CRRT(−) subgroups (27.81 nmol/mL vs. 26.62 nmol/mL, respectively, *p* = 0.506) at ICU admission; there were also no significant differences in citrulline concentrations between the CRRT((+)) and CRRT(−) subgroups on days 3, 5, 7, and 10 (*p* > 0.05). Similar results were obtained for I-FABP, with no differences in I-FABP concentrations between the CRRT((+)) and CRRT(−) subgroups at ICU admission (821.78 pg/mL vs. 651.33 pg/mL, *p* = 0.464). There were also no significant differences in I-FABP concentrations between the CRRT((+)) and CRRT(−) subgroups on days 3, 5, 7, and 10 (*p* > 0.05).

We also assessed whether liver dysfunction affects citrulline and I-FABP levels. On admission to the ICU, 36 patients had no symptoms of liver dysfunction with normal bilirubin levels (<1.2 mg/dL) and 22 patients had bilirubin levels above normal range. Citrulline concentrations were similar in both subgroups (26.46 nmol/mL IQR 19.16–29.93 vs. 28.84 nmol/mL, IQR 24.52–33.30, respectively, *p* = 0.273). I-FABP concentrations were also similar in both subgroups (729.70 pg/mL, IQR 238.00–1216.33 vs. 719.66 pg/mL, IQR 293.00–2740.66, respectively, *p* = 0.696). There were also no significant differences in citrulline and I-FABP concentrations between these subgroups on days 3, 5, 7, and 10 (*p* > 0.05).

The *p*-value shows the differences between groups on corresponding days; a logarithmic scale was used to plot the data. The box plots represent the median values (midpoint) with upper and lower quartiles (box); the whiskers represent the minimum and maximum values.

### 3.2. The Relationship between AGI Score and Biomarker Levels

All septic patients were assessed using the 4-point AGI score on ICU admission (day 1) and on days 3, 5, 7, and 10. A detailed description of the AGI score is provided below in the Section 2. On ICU admission, 69% of patients were graded with AGI I (indicating that the function of the digestive tract is partially impaired, and that patients are at a risk of developing GI dysfunction); 31% were graded with AGI II (indicating the development of GI dysfunction). GI failure was not found in any of the patients on admission to the ICU; therefore, none were classified as AGI III or IV. In the following days, the number of patients with AGI score III (indicating GI failure) increased up to 2%, 29%, 47%, and 50% on day 5, 7, and 10, respectively; none of the patients were classified as AGI IV. Feeding intolerance was 18%, 31%, 29%, 29%, and 23% on days 1, 3, 5, 7, and 10, respectively. Enteral nutrition was administered to 26%, 52%, 57%, 66%, and 61% of patients on days 1, 3, 5, 7, and 10, respectively. In the control group, all patients on admission to the ICU were qualified as AGI I because the operation itself is associated with the risk of developing GI dysfunction. All patients in the control group received a standard postoperative diet after extubation and no enteral tube feeding or parenteral feeding was used in this group; no patient from the control group developed GI dysfunction (AGI II) or GI failure (AGI III).

To assess the relationship between AGI scores and biomarkers level in sepsis, the changes in citrulline and I-FABP levels over time were analyzed in three subgroups, according to highest AGI score calculated during ICU stay: AGI I as the highest calculated score occurred in 20 patients, AGI II in 19, and 19 patients developed severe GI damage and were diagnosed with GI failure (AGI score III). The level of citrulline significantly decreased over time in patients with AGI III (*p* = 0.027), while it did not change significantly in patients with AGI I (*p* = 0.561) and AGI II (*p* = 0.406). The level of I-FABP did not change significantly over time in patients with AGI I (*p* = 0.063), AGI II (*p* = 0.062), and AGI III (*p* = 0.157). We than compared biomarker levels between AGI I vs. AGI III and AGI II vs. AGI III at each time point; results are shown in Table 2. Citrulline levels were significantly reduced in the AGI III subgroup when compared to AGI II throughout the study, while the difference between the AGI III and AGI I subgroups was significant only at day 10. Similar associations were not observed with I-FABP (Table 2).

**Figure 2:**
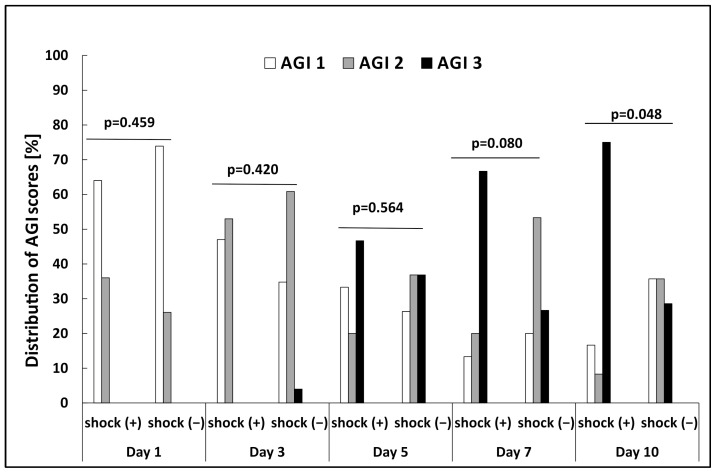
Distribution of the Acute Gastrointestinal Injury (AGI) scores among sepsis patients with and without shock calculated on days 1, 3, 5, 7, and 10.

Next, to assess the effect of septic shock on intestinal cell damage, we compared the distribution of AGI scores between patients with and without septic shock: the distributions of the scores were comparable between septic patients with and without shock on day 1, 3, 5, and 7 of ICU stay. At the end of observation, distributions of the AGI scores differed significantly and most of the patients (75%) with shock were graded with AGI III on day 10. In contrast, in the non-shock group, only 29% of patients on day 10 had AGI III (Figure 2).

### 3.3. Biomarker Levels as a Tool for Predicting the Development of GI Failure

Citrulline had the ability to predict the development of GI failure with an AUC of 0.738 (95% CI 0.607–0.869, *p* < 0.001). The optimal cut-off value for the baseline citrulline level was 27.03 ng/mL, with sensitivity of 78.9% and specificity of 68.8%. The I-FABP did not have the ability to predict the development of GI failure (*p* > 0.05).

**Figure 3:**
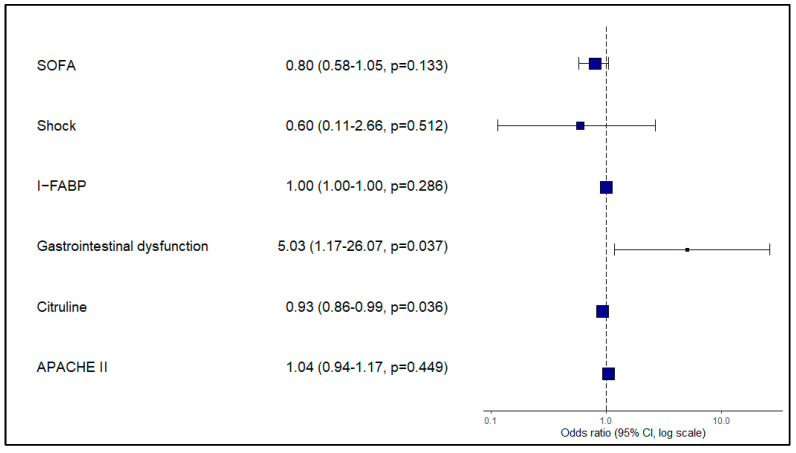
Forest plot of the multivariate logistic regression analysis for predicting the development of gastrointestinal failure during ICU stay (odds ratio, 95% CI, *p*-value are provided). Parameters were calculated on admission to the ICU. (abbreviations) SOFA, Sequential Organ Failure Assessment; I-FABP, intestinal fatty acid binding protein; APACHE II, Acute Physiology and Chronic Health Evaluation II; CI, confidence interval.

In addition, a multivariable logistic regression analysis was performed to create a model predicting the development of GI failure (i.e., AGI score III) during ICU stay in the analyzed cohort. The aim was to check whether the biomarkers measured on admission to the ICU (citrulline, I-FABP) and covariates calculated at ICU admission (APACHE II and SOFA scores, the presence of septic shock, and the presence of GI dysfunction (i.e., AGI score II) at the time of admission to the ICU) predict the development of GI failure during ICU stay. The fitted model reached an accuracy of 0.72 (95% CI 0.60–0.84), a sensitivity of 0.87 (95% CI 0.78–0.96), and a specificity of 0.42 (95% CI 0.29–0.55), when the threshold was set to 0.5. The ROC AUC for the model was 0.77 (95% CI 0.64–0.89) indicating decent discriminative power. The analysis showed that only two out of six predictors were statistically significant: the presence of GI dysfunction at ICU admission (*p*-value = 0.037) and the level of citrulline (*p*-value = 0.036). The results can be interpreted as follows: (1) lower levels of citrulline slightly increased the risk of developing GI failure, and (2) patients with GI dysfunction at the time of admission to the ICU had a 5-fold higher risk of developing GI failure during their stay in the ICU. Figure 3 shows a forest plot with details of the odds ratios for each predictor.

### 3.4. AGI Score, Biomarkers, and 28-Day Mortality

**Figure 4:**
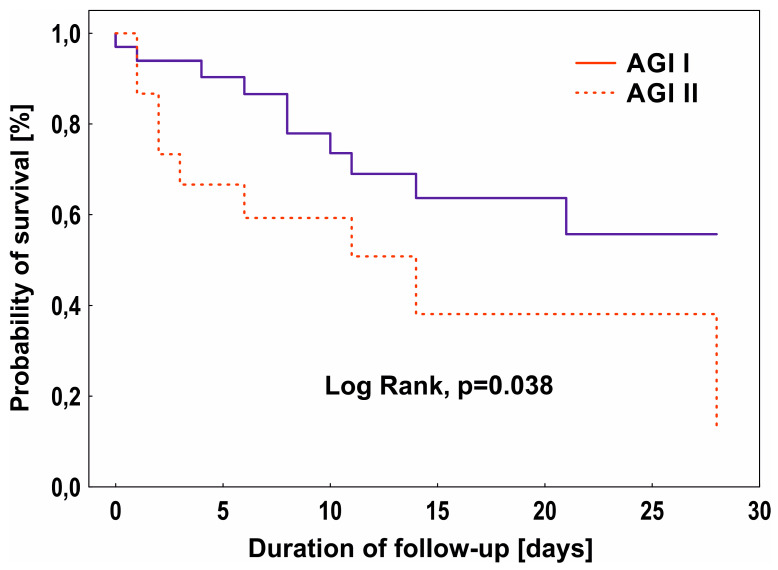
Kaplan–Meier curves stratified by the Acute Gastrointestinal Injury (AGI) scores (AGI I vs. AGI II) calculated in patients with sepsis at Intensive Care Unit (ICU) admission for 28 day mortality. *p*-value was for differences across the AGI scores by log-rank test.

On ICU admission, 69% of patients were graded with AGI I and 31% with AGI II. GI failure was not found in any of the patients on admission to the ICU; therefore, none were classified as AGI III. There was a significant difference in 28 day survival between groups with different AGI scores calculated at ICU admission: an AGI II score was associated with a worse prognosis when compared to AGI I (log-rank test *p* = 0.038). Figure 4 shows the Kaplan–Meier curves stratified on the AGI score for 28 day mortality in the studied population. However, there were no differences in citrulline or I-FABP levels between patients who died and those who survived throughout the study period (*p* > 0.05).

## 4. Discussion

In our study, we assessed changes in citrulline and I-FABP concentrations in a well-defined group of ICU patients diagnosed with bacterial sepsis/septic shock. We found significant alterations in citrulline and I-FABP levels during sepsis, with decreased citrulline levels possibly indicating a reduction in functional enterocyte mass and elevated I-FABP levels suggesting enterocyte damage. In addition, citrulline concentrations were significantly lower in patients with septic shock, reflecting shock-related intestinal ischemia and damage to enterocytes and the intestinal barrier. Our results showed that plasma level of citrulline and the diagnosis of GI dysfunction at the time of admission to the ICU were associated with significantly higher risk of developing GI failure during ICU stay in the analyzed cohort. The predictive ability of baseline levels of citrulline and I-FABP for 28 day mortality was low; therefore, these biomarkers do not seem to be good candidates as independent predictors of mortality in our sepsis patient population. On the other hand, the AGI score calculated in septic patients on the first day of ICU admission was a strong predictor for 28 day mortality.

Various biomarkers have been studied in recent years to assess intestinal damage in critically ill patients, and I-FABP and citrulline appear to be the most promising [29,30,31]. I-FABP, a protein responsible for binding fatty acids in the intestine, is produced by the intestinal epithelium and released upon intestinal villi ischemia and damage [32,33]. Its importance as a clinical tool has been demonstrated in type 2 diabetes, necrotizing enterocolitis, mesenteric ischemia, and abdominal trauma [34,35,36]. Citrulline is an amino acid synthesized mainly in the small-bowel epithelium [17]. Intestinal damage leads to a decrease in the enterocyte mass, and thus to a reduction in synthesis of the amino acid; this results in a low concentration of citrulline in plasma [37]. Citrulline has also turned out to be a useful predictive tool in chronic inflammatory intestinal diseases [38].

The application of citrulline and I-FABP as biomarkers of intestinal damage was also evaluated in animal models of sepsis and in patients with sepsis and septic shock. In an animal model of sepsis, damage to the intestinal mucosa was evident with changes in intestinal villi length and mucosal thickness. Consequently, the level of citrulline was lower and I-FABP higher than in the control group (animals without sepsis), reflecting changes in both the number and function of intestinal epithelial cells; citrulline in animals with sepsis decreased as a result of reduced production by damaged enterocytes and I-FABP increased in the bloodstream in the presence of intestinal ischemia. Thus, the measurement of both citrulline and I-FABP may be useful as a non-invasive tool for the diagnosis of sepsis-induced intestinal damage [39]. To date, only a few studies have been conducted evaluating the usefulness of citrulline as a marker of intestinal damage and prognosis in a population of patients with sepsis. In a pediatric population of critically ill patients, a decrease in citrulline concentration was associated with worse prognosis (increased acute phase parameters, longer mechanical ventilation, and a longer ICU stay) [40]. Another study on a general population of ICU patients by Piton et al. showed that low plasma citrulline levels on ICU admission were associated with high CRP, higher rates of nosocomial infections, and 28 day mortality [21]. Patients with septic shock accounted for 7% of the population studied by Piton, but the mean levels of citrulline concentrations in this group were <20 nmol/mL throughout the observation period. In our study, all patients were diagnosed with sepsis and patients with septic shock accounted for 52%. We hypothesized that septic shock-related intestinal ischemia and hypoperfusion may have resulted in damage to the intestinal barrier and a reduction in enterocyte mass. In fact, plasma citrulline levels were significantly reduced in these patients when compared to the values measured in the non-shock septic group, confirming our hypothesis. It is worth noting that in the entire study group of patients with sepsis (both with and without septic shock), the levels of citrulline were already lower on admission to the ICU when compared to previously reported values in healthy subjects; the optimal cut-off value for the baseline citrulline level to predict the development of GI failure was 27.03 ng/mL [41].

I-FABP is a protein that is expressed almost exclusively in enterocytes and is released from damaged cells into the bloodstream [33,42]. Therefore, elevation in plasma I-FABP may indicate damage to the intestinal wall. In healthy people, enterocytes undergo apoptosis without extracellular release of intracellular proteins including proteolytic enzymes and other DAMPs (damage-associated molecular patterns) and I-FABP plasma levels are very low or even undetectable [43]. In a study by Haan et al., a median level of 87 pg/mL was established based on measurements of plasma collected from 57 healthy volunteers [44]. In our study, both the septic group and the ICU control group had I-FABP levels well above this reference value. We could also confirm that plasma levels of citrulline and I-FABP were associated with septic shock. On each of the study days, our patients with septic shock had significantly lower citrulline levels than those without shock, but I-FABP was significantly higher only on days 1 and 3 in the septic shock group. This may be the result of treatment leading to attenuation of the systemic inflammatory response to infection and thus less damage to the enterocytes. Intestinal damage, as indicated by low citrulline levels and high I-FABP levels, has been shown to be associated with shock [20,45]. Derikx et al. showed a direct relationship between splanchnic hypoperfusion, measured with gastric mucosal tonometry, and intestinal mucosal damage, confirmed by high plasma I-FABP levels in the early phase of abdominal sepsis [24]. The release of cytosolic proteins into the bloodstream as a result of intestinal cell damage has also been reported in COVID-19 patients complicated by septic shock, who had higher I-FABP levels when compared to non-shock patients [27]. This may suggest that the release of I-FABP from damaged enterocytes during septic shock is independent of the type of causative pathogen and occurs in both bacterial and viral sepsis; further research is needed to confirm this observation.

The Acute Gastrointestinal Injury (AGI) scale is one of the few useful tools to assess the degree of intestinal injury in ICU settings. It was developed by ESICM in 2012 [15] and validated in 2017 [2]. Many studies have used the AGI score as a major indication of intestinal dysfunction and failure in variety of clinical settings, e.g., in critically ill COVID-19 patients, in patients with foodborne sepsis, and to evaluate enteral feeding protocol in critically ill patients and gut rest strategy [46,47,48,49,50]. In the present study, biomarker levels measured on days 1, 3, 5, 7, and 10 were assessed by comparison with the severity of intestinal dysfunction determined by the AGI score on respective days. Our results confirm that declining citrulline levels are associated with an increased risk of GI dysfunction and failure in patients with sepsis, similar to what was shown in the IN-PANCIA study and another study by Teng et al. in a general ICU population [19,51]. Unlike citrulline, changes in I-FABP levels were not associated with the AGI score in sepsis patients on any study day. A similar result was shown by Li et al., where a high level of I-FABP did not increase the odds of any AGI score in a general population of critically ill ICU patients [31]. In addition, we attempted to create a model predicting the development of GI failure during ICU stay. According to the results of multivariate logistic regression analysis, an elevated AGI score already at admission to the ICU (indicating GI dysfunction) and a decreased citrulline concentration were associated with a significantly higher risk of developing GI failure during ICU stay.

The distribution of AGI scores in our study between patients with and without septic shock was comparable on the first days of the study and significantly different at the end of observation. Most patients with shock had AGI score III (75%); among those who were not in shock, only 29% had AGI III on day 10. This indicates severe intestinal injury, manifested by persistent feeding intolerance despite treatment. Our results are consistent with previously published results by Sun et al., which showed that higher scores of AGI were associated with worse clinical variables, higher rates of septic shock, and higher 28 day mortality in a cohort of critically ill COVID-19 patients [47]. The relationship between AGI score and ICU outcome was studied by Klanovicz et al. [52], who found that AGI III present within the first 48 h after ICU admission was a significant risk factor for ICU mortality in patients with septic shock undergoing mechanical ventilation. In septic and septic shock patients, AGI score on admission turns out to be a good 28 day survival predictive tool. We found that AGI II score calculated at ICU admission was associated with a significantly worse prognosis of survival when compared to AGI I. As confirmed by a recent meta-analysis, the incidence of acute GI injury in critically ill patients is high (40%) and is associated with higher mortality; therefore, ESICM-initiated stratification may facilitate the identification and treatment of patients at risk of acute GI injury, and consequently better treatment outcomes [53].

## 5. Limitations

We are aware of our study limitations. First, the study was conducted in a single center with a relatively small patient population and needs to be validated in a much larger cohort of patients with sepsis and septic shock and critically ill ICU patients without infection. In a multivariate logistic regression analysis performed to model GI failure, the 95% confidence interval for the AGI score calculated at ICU admission is wide, which can be attributed to the small size of the dataset. Future research on a larger data set is needed. However, it should be emphasized that our study group is well defined and limited to sepsis and septic shock of bacterial etiology. Also, the control group is small but is well defined as a group with no prior infection and no infectious complications during the ICU stay. Second, it is still not clear how to interpret low citrulline results in patients with sepsis/septic shock: does low citrulline value require a specific therapeutic approach, and what value of citrulline should be considered as the cut-off point for supplementation? These issues need to be addressed in future studies.

## 6. Conclusions

GI dysfunction in septic patients on the first day of ICU admission, confirmed by a worse AGI score, is a predictor for 28 day mortality. In addition, an elevated AGI score on ICU admission, together with a decrease in citrulline levels, were associated with a higher risk of developing GI failure during ICU stay. Intestinal wall ischemia associated with hypoperfusion in septic shock may result in damage to the intestinal barrier and a reduction in enterocyte mass and function, as indicated by a significant decrease in plasma citrulline and I-FABP in the first days upon ICU admission. However, it should be noted that the levels of citrulline and I-FABP may be elevated in many conditions associated with disorders of the GI tract, kidneys, or liver, which may occur simultaneously in patients with sepsis.

## Figures and Tables

**Table 1:** Baseline characteristics of patients.

Parameter	Control	Septic Patients		
	*N* = 10	Shock (+), *N* = 30	Shock (−), *N* = 28	p *
Age, years	67.5 (61.0–70.0)	66.0 (60.0–73.0)	61.5 (55.5–72.5)	0.198
Male, n (%)	7.0 (70.0)	18.0 (60.0)	16.0 (57.0)	0.825
BMI, kg/m^2^	27.0 (24.0– 29.1)	27.8 (25.2–30.9)	26.1 (24.3–29.7)	0.171
APACHE II score	10.5 (10–11)	28.0 (24.0–32.0)	24 (18–28)	0.046
SOFA score	2 (1–3)	10 (8.0–13.0)	9 (8–12)	0.388
ICU admission *n* (%):				0.360
Medical	0.0	18.0 (60.0)	20.0 (71.0)	
Surgical	10.0 (100.0)	12.0 (40.0)	8.0 (29)	
Lactate [mmol/L]	0.9 (0.7–1.1)	4.6 (2.6–8.2)	1.7 (1.2–1.8)	<0.001
PLT [10^3^/uL]	159.0 (137.0–181.0)	210.0 (124.0–309.0)	174.5 (120.0–364.5)	0.803
Fibrinogen [g/L]	2.9 (3.6–6.5)	4.6 (3.6–6.0)	5.6 (3.7–6.6)	0.481
D-dimer [mg/L]	0.7 (0.4–2.1)	6.2 (3.9–15.7)	6.2 (2.7–10.1)	0.395
WBC [10^3^/uL]	12.6 (11.2–16.1)	17.1 (11.3–27.6)	13.3 (9.1–21.1)	0.543
CRP [mg/L]	61.6 (35.5–106.7)	194.6 (104.1–328.4)	255.6 (164.5–344.5)	0.358
PCT [ng/mL]	0.1 (0.0–0.1)	10.6 (3.6–34.2)	8.7 (3.5–23.4)	0.528
Treatment *n* (%):				
CRRT	0.0	12.0 (40.0)	6.0 (21.0)	0.126
Mechanical ventilation	10.0 (100)	19.0 (63.0)	22.0 (79.0)	0.202
ICU LOS [day]	2.0 (2.0–3.0)	7.5 (2.0–17.5)	11 (5.0–21.0)	0.093
Mortality, 28 days (%)	0.0	50.0	25.0	0.049

Values are presented as the median and the interquartile range or as frequencies with percentages; * the *p*-value represents the difference between patients with and without septic shock. Abbreviations: BMI, Body Mass Index; APACHE II, Acute Physiology and Chronic Health Evaluation II; SOFA, Sequential Organ Failure Assessment; ICU, Intensive Care Unit; PLT, Platelets; WBC, White Blood Cells; CRP, C-Reactive Protein; PCT, Procalcitonin; CRRT, Continuous Renal-Replacement Therapy; LOS, Length of Stay.

**Table 2:** The relationship between the AGI score and the level of biomarkers. During ICU stay, AGI I was the highest calculated result in 20 patients, AGI II in 19 patients, and in 19 patients severe damage to the GI tract occurred and GI failure was diagnosed (AGI III).

	Day 1	Day 3	Day 5	Day 7	Day 10
			Citrulline [nmol/mL]		
AGI I	27.36	25.03	26.72	24.11	33.81
	(13.36–30.58)	(23.30–30.92)	(23.63–46.54)	(23.42–24.40)	(27.55–35.22)
AGI II	31.00	27.56	32.66	29.20	29.40
	(27.26–33.30)	(25.62–36.87)	(25.62–36.87)	(21.33–31.45)	(23.84–47.95)
AGI III	24.59	23.42	22.14	21.50	18.46
	(12.30–26.85)	(12.64–27.47)	(9.92–26.81)	(10.32–25.88)	(8.88–26.33)
* p	0.244	0.197	0.053	0.205	0.037
*# p*	<0.001	0.018	0.005	0.027	0.022
			I-FABP [pg/mL]		
AGI I	476.61	400.71	546.00	493.21	2125.36
	(146.33–1095.36)	(303.84–812.14)	(155.27–1633.57)	(484.00–610.67)	(694.00–3204.29)
AGI II	956.33	414.67	421.33	1146.33	937.87
	(429.67–2191.00)	(254.67–1002.54)	(286.33–2432.86)	(431.00–1443.00)	(373.00–1139.67)
AGI III	832.57	283.76	416.78	550.35	698.10
	(125.80–1148.27)	(198.17–831.07)	(173.33–690.00)	(322.33–1673.00)	(225.71–1479.29)
* p	0.693	0.424	0.793	0.753	0.231
*# p*	0.293	0.447	0.451	0.887	0.650

Values are presented as the median and the interquartile range; * *p*-value represents the difference in biomarker levels in AGI I vs. AGI III; *# p*-value represents the difference in biomarker levels in AGI II vs. AGI III. Abbreviations: AGI, Acute Gastrointestinal Injury; I-FABP, Intestinal Fatty Acid Binding Protein.

## Data Availability

The data presented in the study are available on request from the corresponding author. The data have not been made publicly available because they contain information that could compromise the privacy of the study participants.
